# Application of a nomogram model for the prediction of 90-day poor outcomes following mechanical thrombectomy in patients with acute anterior circulation large-vessel occlusion

**DOI:** 10.3389/fneur.2024.1259973

**Published:** 2024-01-19

**Authors:** Xia Li, Chen Li, Ao-fei Liu, Chang-chun Jiang, Yi-qun Zhang, Yun-e Liu, Ying-ying Zhang, Hao-yang Li, Wei-jian Jiang, Jin Lv

**Affiliations:** ^1^The PLA Rocket Force Characteristic Medical Center, Beijing, China; ^2^Department of Neurology, Baotou Center Hospital, Neurointerventional Medical Center of Inner Mongolia Medical University, Institute of Cerebrovascular Disease in Inner Mongolia, Inner Mongolia, China; ^3^Department of Psychiatric Specialty, Capital Medical University, Beijing, China

**Keywords:** mechanical thrombectomy (MT), large-vessel occlusion (LVO), acute ischemic stroke (AIS), poor outcome, nomogram, prognostic predictive model

## Abstract

**Background:**

The past decade has witnessed advancements in mechanical thrombectomy (MT) for acute large-vessel occlusions (LVOs). However, only approximately half of the patients with LVO undergoing MT show the best/independent 90-day favorable outcome. This study aimed to develop a nomogram for predicting 90-day poor outcomes in patients with LVO treated with MT.

**Methods:**

A total of 187 patients who received MT were retrospectively analyzed. Factors associated with 90-day poor outcomes (defined as mRS of 4–6) were determined by univariate and multivariate logistic regression analyzes. One best-fit nomogram was established to predict the risk of a 90-day poor outcome, and a concordance index was utilized to evaluate the performance of the model. Additionally, 145 patients from a single stroke center were retrospectively recruited as the validation cohort to test the newly established nomogram.

**Results:**

The overall incidence of 90-day poor outcomes was 45.16%, affecting 84 of 186 patients in the training set. Moreover, five variables, namely, age (odds ratio [OR]: 1.049, 95% CI [1.016–1.083]; *p* = 0.003), glucose level (OR: 1.163, 95% CI [1.038–1.303]; *p* = 0.009), baseline National Institute of Health Stroke Scale (NIHSS) score (OR: 1.066, 95% CI [0.995–1.142]; *p* = 0.069), unsuccessful recanalization (defined as a TICI grade of 0 to 2a) (OR: 3.730, 95% CI [1.688–8.245]; *p* = 0.001), and early neurological deterioration (END, defined as an increase of ≥4 points between the baseline NIHSS score and the NIHSS score at 24 h after MT) (OR: 3.383, 95% CI [1.411–8.106]; *p* = 0.006), were included in the nomogram to predict the potential risk of poor outcomes at 90 days following MT in LVO patients, with a C-index of 0.763 (0.693–0.832) in the training set and 0.804 (0.719–0.889) in the validation set.

**Conclusion:**

The proposed nomogram provided clinical evidence for the effective control of these risk factors before or during the process of MT surgery in LVO patients.

## Introduction

Due to its well-established high efficiency and relative safety, mechanical thrombectomy (MT) has been considered the first-line strategy for managing acute anterior circulation (AC)-large-vessel occlusion (LVO) for the past several years (1–5). MT in patients with LVO-induced stroke, even with an extended time window of 24 h (6, 7), has demonstrated improved recanalization rates (58.7 to 88%), amelioration of functional outcomes, and reduced disability rates at 90 days (8). Despite these beneficial effects, more than half of the patients (51–56.7%) still exhibit a non-independent functional outcome (modified Rankin Scale [mRS] score of ≥3 at 90 days) after MT (6, 7, 9). In addition, 37.1% of patients who underwent MT were severely disabled for life (mRS score of ≥4 at 90 days), and even with successful recanalization following MT, only 27% of the patients were disability-free at 90 days. The definition of non-independent functional outcomes after MT has been a topic of debate, with most studies suggesting that non-functionally independent outcomes include mainly moderate disability, severe disability, very severe disability, and death. However, moderate disability is characterized by the ability to walk with help or assistance, which, in reality, is an acceptable functional outcome after MT for acute LVO. Furthermore, the median mRS score for the 90-day clinical outcome in most RCT studies comparing the findings with medication was 3, which was closer to the mRS score of 2 rather than 4 in clinical practice (10). Therefore, more clinical attention should be directed toward the poor outcomes that impose an unbearable burden on stroke patients, such as the inability to walk and to look after themselves with necessary help, and even worse scenarios.

At present, to ensure the maximum benefit of MT for LVO stroke patients, a large number of neurologists are committed to exploring the risk factors for unfavorable outcomes (a 90-day mRS score of 3–6) after MT by using various clinical predictive models and scoring systems for the accurate prediction and precise assessment of the surgical procedure (11, 12). While a vast majority of such studies (13–16) have focused on non-independent functional outcomes (mRS score of 3–6) as unfavorable outcomes at 90 days following MT for acute stroke, few studies have addressed the 90-day poor outcomes (mRS score of 4–6) in AC-LVO patients who underwent MT, and studies reporting the risk factors for poor outcomes in this patient population are even more scarce.

Thus, the present study aimed to identify the clinical risk factors for 90-day poor outcomes (mRS score of 4–6) after MT in patients with acute AC ischemic stroke, to develop a nomogram model for predicting the probability of poor outcomes at 90 days in this population, and to validate the performance of this nomogram using an external single-center dataset.

## Methods

### Study design and population

**Figure 1:**
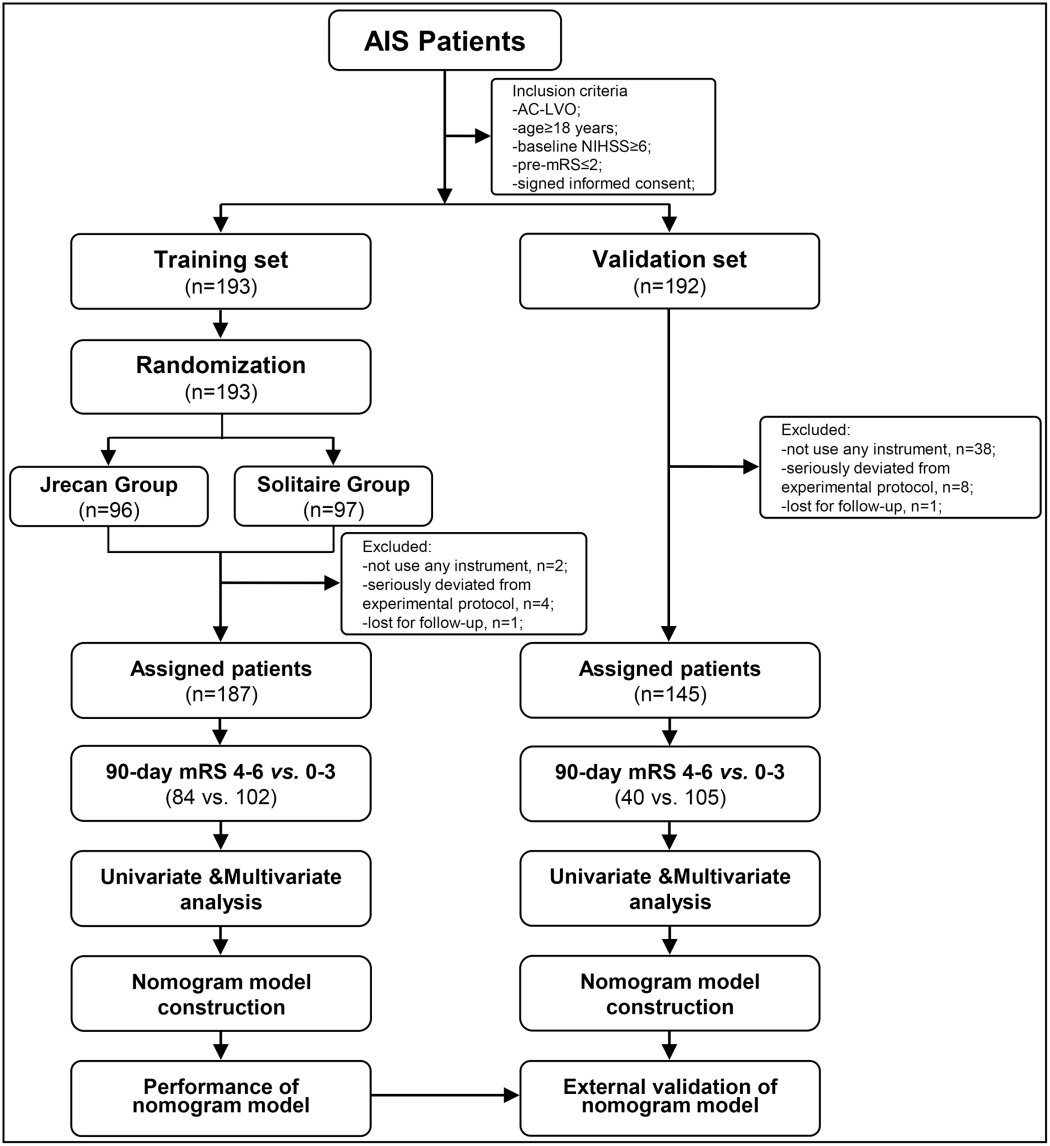
A flowchart of eligible patients with acute large-vessel occlusions in the anterior circulation. AIS, acute ischemic stroke; AC-LVO, anterior circulation large-vessel occlusion; mRS, modified Rankin Scale; NIHSS, National Institute of Health Stroke Scale.

This study was a retrospective investigation of the prospectively collected data from the Jrecan trial between 1 March 2018 and 30 June 2019, which was named as the training set. The Jrecan trial was a prospective, multicenter, randomized, non-inferiority clinical trial comparing the efficacy and safety of two MT stents (the Jrecan revascularization device and the Solitaire device) in patients with AC-LVO stroke. The trial involved 16 comprehensive stroke centers in China. Each center handled more than 50 cases of MT annually, and each neuro-interventionalist performed at least 10 MT procedures per year. The Jrecan trial was approved by the ethics committees of the participating centers and was duly registered with the Chinese Clinical Trial Registry, http://www.chictr.org.cn/showproj.aspx?proj=23396, identifier ChiCTR-TOC-17013822. The patient data for the validation set were retrospectively collected from Baotou Center Hospital between January 2013 and December 2020 and approved by the ethics committee of Baotou Center Hospital. Informed consent was obtained from the participants or their legal representatives. The flowchart of the patient profile is shown in Figure 1.

Patients were enrolled if they met the following criteria: (1) age ≥ 18 years; (2) diagnosed with acute ischemic stroke (AIS) within 8 h of symptom onset; (3) baseline admission National Institute of Health Stroke Scale (NIHSS) score of ≥6; (4) modified Rankin Scale (mRS) score prior to stroke ≤2; and (4) proximal intracranial large artery occlusion in the AC confirmed by digital subtraction angiography. All participants and their legal representatives provided written informed consent.

### Procedures

Stroke patients underwent mechanical thrombectomy under either conscious sedation or general anesthesia within 8 h of symptom onset, and those eligible for thrombolysis received intravenous recombinant tissue plasminogen activator (IV-rtPA) within 4.5 h of stroke onset. Patients were randomly assigned to receive either the Jrecan or Solitaire^™^ stent for MT in a 1:1 ratio. The goal of thrombectomy was to achieve a modified thrombolysis in cerebral infarction (mTICI) grade of 2b or 3. Salvage therapies, such as intra-arterial thrombolysis, balloon angioplasty, or stent implantation, were performed if recanalization of the target vessel was not achieved after three passes of thrombectomy. MT was considered to have failed if the patients required rescue therapy. The time from stroke onset to admission was documented for all cases. All patients were followed up for 90 days after MT.

### Outcome measures

Functional outcomes were determined by measuring the mRS score, and poor outcomes were defined as an mRS score of 4–6 at 90 days. Successful recanalization was defined as a TICI grade of 2b or 3. Early neurological deterioration (END) was defined as an increase of ≥4 points between the baseline NIHSS score and the NIHSS score at 24 h after MT.

### Statistical analysis

Statistical analysis was performed with SPSS version 22.0 (IBM Corporation, Armonk, New York, United States) or R version 4.1.2 software (http://www.R-project.org, Foundation for Statistical Computing, Vienna, Austria). Categorical variables were presented as numbers (percentage, %). Continuous variables were expressed as mean (standard deviation, [SD]) or median (interquartile range, [IQR]) and were dichotomized into categorical variables using the median as a cutoff value.

Univariable logistic regression analysis was first performed to identify potential predictive factors of 90-day poor outcomes (mRS score of 4–6) in patients receiving MT, with the Mann–Whitney U-tests for continuous variables and Fisher exact or *χ*^2^ tests for categorical variables. Then, variables with *p-*values<0.05 in the univariate analysis were included in the multivariate logistic regression analysis. Based on the results of the multivariable logistic regression analysis, a nomogram model for predicting poor outcomes at 90 days was constructed, which determined the likelihood of poor outcomes by assigning each predictor a score of 0–100. The total score of each patient was obtained by summing the individual scores of each predictor, and then, the individual probabilities of poor outcomes ranging from 0 to 100% were derived. The discriminative power of the nomogram was assessed using Harrell’s concordance index (C-index). The performance of the nomogram was externally validated in a validation set. A calibration plot was drawn to verify the fit between the actual outcomes and the poor outcomes predicted by the nomogram. Decision curve analyzes were used to discriminate between the net benefit and the probability of poor outcomes in patients with LVO undergoing MT.

## Results

### General characteristics of participants

**Figure 2:**
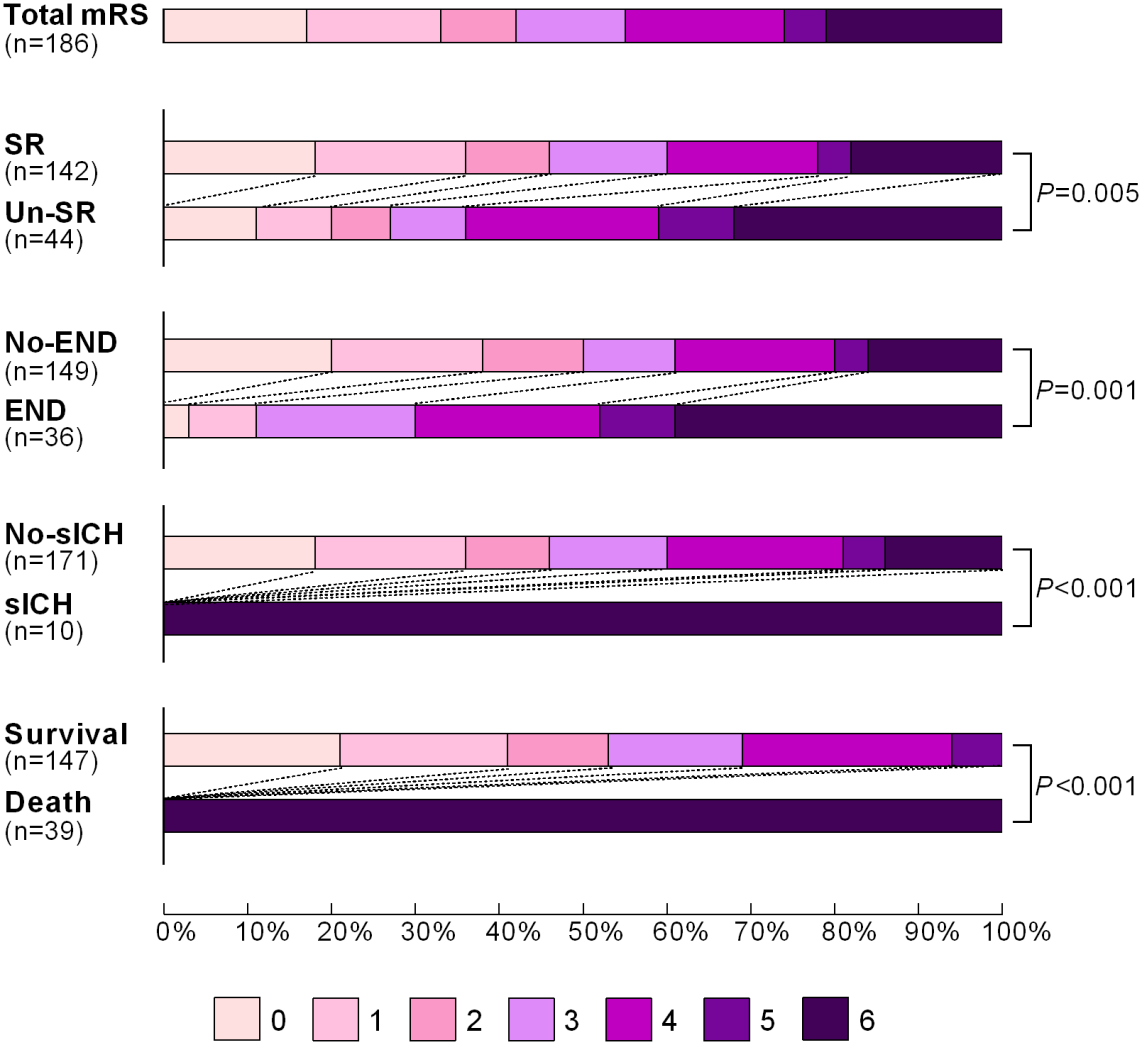
Distribution of the modified Rankin Scale (mRS) score of patients with successful recanalization vs. unsuccessful recanalization, END vs. no-END, sICH vs. no-sICH, and survival vs. death at 90 days after mechanical thrombectomy. The mRS scores ranged from 0 to 6, with the higher scores indicating a more severe disability. SR, successful recanalization; Un-SR, unsuccessful recanalization; END, early neurological deterioration; sICH, symptomatic intracerebral hemorrhage.

In the training set, a total of 187 patients were enrolled in the Jrecan trial, and 1 patient was lost to follow-up after discharge (Figure 1). The mean age of the patients was 66 years (IQR, 58–74 years), and 56.7% (106/187) of the patients were men. The median baseline NIHSS score was 14 (IQR, 11–18), and the median ASPECT score was 9 (IQR, 8–10). The median onset to admission time was 180 min (IQR, 110–268 min), and 32.6% (61/187) of the patients received IV-rtPA. The rate of unsuccessful recanalization was 24.1% (45/186). Poor outcomes were observed in 84 of 186 patients (45.2%) at 90 days. In terms of complications within 24 h, 19.3% (36/187) of the patients showed early neurological deterioration (END), and 5.3% (10/187) of the patients showed symptomatic intracerebral hemorrhage (sICH). The modified Rankin Scale (mRS) score distributions in patients who underwent successful recanalization versus those who did not, patients who exhibited END or sICH versus those who did not, and those who had survived or died at 90 days were compared. The results are shown in Figure 2. The survival status of the patients with successful recanalization versus unsuccessful recanalization, END versus no-END, sICH versus no-sICH, and poor outcomes (90-day mRS score of 4–6) versus acceptable functional recovery (90-day mRS score of 0–3) was illustrated using the K–M curves, and statistically significant differences were observed between the groups (*p* < 0.05) (Supplementary Figure S1). Baseline patient characteristics and procedural parameters are listed in Supplementary Table S1.

### Predictors of 90-day poor outcomes in patients with AC-LVO undergoing MT

The baseline characteristics of patients with AC-LVO in the training and validation sets grouped by 90-day outcomes (mRS score, 4–6 vs. 0–3) are shown in Table 1. Continuous variables, including age, glucose level, systolic blood pressure, diastolic blood pressure, baseline NIHSS score, ASPECTS score, and onset to admission time, are presented as median with IQR. A univariable analysis identified age, glucose level, baseline NIHSS score, unsuccessful recanalization, and END as potential predictors (with *p* < 0.05), all of which were finally entered into the multivariable logistic regression model for predicting the probability of a 90-day poor outcome following MT (Table 1). In the multivariable analysis, age (OR: 1.049 [95% CI: 1.016–1.083]; *p* = 0.003), glucose level (OR: 1.163 [95% CI: 1.038–1.303]; *p* = 0.009), unsuccessful recanalization (OR: 3.730 [95% CI: 1.688–8.245]; *p* = 0.001), and the occurrence of END (OR: 3.383 [95% CI: 1.411–8.106]; *p* = 0.006) were associated with a poor outcome at 90 days following MT in the training set (Table 2), while age (OR: 1.051 [95% CI: 1.008–1.095]; *p* = 0.020), baseline NIHSS (OR: 1.187 [95% CI: 1.055–1.335]; *p* = 0.004), and the occurrence of END (OR: 3.548 [95% CI: 1.068–11.787]; *p* = 0.039) were identified as independent predictors of 90-day poor outcomes in the validation set (Table 2).

**Table 1:** Univariable analysis of the factor associated with 90-day poor outcomes (mRS scores of 4–6) following mechanical thrombectomy (MT) in patients with anterior circulation large-vessel occlusion (AC-LVO) in the training set and the validation set.

Variables	Statistics	Training set (*n* = 187)		Validation set (*n* = 145)	
		mRS ≥ 4 (*n* = 84)	mRS ≤ 3 (*n* = 102)	mRS ≥ 4 (*n* = 40)	mRS ≤ 3 (*n* = 105)
**Demographics**					
Age	Median, IQR	68 (64 ~ 77)	64 (54 ~ 70)	70 (62 ~ 75)	59 (53 ~ 70)
	*p*-value	<0.001		0.001	
Sex	Male	51.2% (43/84)	60.8% (62/102)	55.0% (22/40)	68.6% (72/105)
	Female	48.8% (41/84)	39.2% (40/102)	45.0% (18/40)	31.4% (33/105)
	*p*-value	0.189		0.126	
**Medical History**					
Smoke habits	Yes	20.2% (17/84)	32.4% (33/102)	20.0% (8/40)	44.8% (47/105)
	No	79.8% (67/84)	67.6% (69/102)	80.0% (32/40)	55.2% (58/105)
	*p*-value	0.064		0.006	
Hypertension	Yes	66.7% (56/84)	53.9% (55/102)	60.0% (24/40)	54.3% (57/105)
	No	33.3% (28/84)	46.1% (47/102)	40.0% (16/40)	45.7% (48/105)
	*p*-value	0.078		0.536	
Diabetes	Yes	28.6% (24/84)	16.7% (17/102)	32.5% (13/40)	21.9% (23/105)
	No	71.4% (60/84)	83.3% (85/102)	67.5% (27/40)	78.1% (82/105)
	*p*-value	0.051		0.187	
Coronary heart disease	Yes	19.0% (16/84)	14.7% (15/102)	40.0% (16/40)	21.9% (23/105)
	No	81.0% (68/84)	85.3% (87/102)	60.0% (24/40)	78.1% (82/105)
	*p*-value	0.429		0.028	
Atrial fibrillation	Yes	52.4% (44/84)	45.1% (46/102)	47.5% (19/40)	22.9% (24/105)
	No	47.6% (40/84)	54.9% (56/102)	52.5% (21/40)	77.1% (81/105)
	*p*-value	0.323		0.004	
Previous stroke	Yes	14.3% (12/84)	9.8% (10/102)	25.0% (10/40)	17.1% (18/105)
	No	85.7% (72/84)	90.2% (92/102)	75.0% (30/40)	82.9% (87/105)
	*p*-value	0.346		0.284	
**Baseline data**					
Pre-operation mRS	=0	91.7% (77/84)	96.1% (98/102)	85.0% (34/40)	96.2% (100/104)
	≠0	8.3% (7/84)	3.9% (4/102)	15.0% (6/40)	3.8% (4/104)
	*p*-value	0.204		0.018	
Glucose	Median, IQR	7.84 (6.09 ~ 9.66)	6.6 (5.89 ~ 8.22)	7.93 (6.67 ~ 10.11)	6.55 (5.09 ~ 8.17)
	*p*-value	0.003		0.022	
Systolic pressure	Median, IQR	141 (12–6 ~ 160)	138 (122 ~ 151)	150 (140 ~ 160)	142 (130 ~ 158)
	*p*-value	0.086		0.016	
Diastolic pressure	Median, IQR	81 (73 ~ 91.5)	82 (77 ~ 90)	90 (76 ~ 98)	90 (80 ~ 98)
	*p*-value	0.998		0.796	
Baseline NIHSS	Median, IQR	14.5 (12 ~ 19.5)	14 (11 ~ 17)	14 (12.2 ~ 16.8)	12 (9.5 ~ 14.5)
	*p-*value	0.042		<0.001	
ASPECT	Median, IQR	8 (7 ~ 10)	9 (8 ~ 10)	7 (6 ~ 8)	8 (7 ~ 9)
	*p*-value	0.156		0.055	
TOAST	Atheroma	25.0% (21/84)	18.6% (19/102)	32.5% (13/40)	41.9% (44/105)
	Cardioembolic	75.0% (63/84)	81.4% (83/102)	67.5% (27/40)	58.1% (61/105)
	*p*-value	0.292		0.300	
Intravenous thrombolysis	Yes	28.6% (24/84)	35.3% (36/102)	25.0% (10/40)	31.4% (33/105)
	No	71.4% (60/84)	64.7% (66/102)	75.0% (30/40)	68.6% (72/105)
	*p*-value	0.329		0.449	
Anesthesia methods	GA	41.7% (35/84)	52.9% (54/102)	5.0% (2/40)	4.8% (5/105)
	Local	58.3% (49/84)	47.1% (48/102)	95.0% (38/40)	95.2% (100/105)
	*p*-value	0.126		0.952	
Occlusion location	M1	67.9% (57/84)	75.5% (77/102)	50.0% (20/40)	62.9% (66/105)
	ICA	32.1% (27/84)	24.5% (25/102)	50.0% (20/40)	37.1% (39/105)
	*p-*value	0.248		0.159	
Unsuccessful recanalization	Yes	33.3% (28/84)	15.7% (16/102)	35.0% (14/40)	15.2% (16/105)
	No	66.7% (56/84)	84.3% (86/102)	65.0% (26/40)	84.8% (89/105)
	*p*-value	0.005		0.009	
Residual severe stenosis	Yes	16.7% (14/84)	17.6% (18/102)	22.5% (9/40)	31.4% (33/105)
	No	83.3% (70/84)	82.4% (84/102)	77.5% (31/40)	68.6% (72/105)
	*p*-value	0.860		0.289	
Remote embolization	Yes	26.2% (22/84)	20.6% (21/102)	–	–
	No	73.8% (62/84)	79.4% (81/102)	–	–
	*p*-value	0.367			
Rescue therapy	Yes	28.6% (24/84)	19.6% (20/102)	10.0% (4/40)	10.5% (11/105)
	No	71.4% (60/84)	80.4% (82/102)	90.0% (36/40)	89.5% (94/105)
	*p*-value	0.152		0.933	
Anticoagulant therapy	Yes	56.0% (47/84)	56.9% (58/102)	30.0% (12/40)	21.9% (23/105)
	No	44.0% (37/84)	43.1% (44/102)	70.0% (28/40)	78.1% (82/105)
	*p*-value	0.901		0.309	
OTA	Median, IQR	190 (120 ~ 269)	186 (119 ~ 270)	190 (117 ~ 271)	189 (131 ~ 280)
	*p*-value	0.887		0.885	
OTP	Median, IQR	311 (229 ~ 392)	300 (230 ~ 375)	329 (266 ~ 377)	352 (276 ~ 452)
	*p*-value	0.890		0.237	
OTR	Median, IQR	449 (334 ~ 515)	408 (323 ~ 480)	434 (395 ~ 501)	432 (356 ~ 544)
	*p*-value	0.458		0.974	
**Complications**					
END	Yes	30.1% (25/83)	10.8% (11/102)	27.5% (11/40)	8.6% (9/105)
	No	69.9% (58/83)	89.2% (91/102)	72.5% (29/40)	91.4% (96/105)
	*p*-value	0.001		0.003	
24 h ICH without END	Yes	27.8% (22/79)	21.6% (22/102)	20.0% (8/40)	23.8% (25/105)
	No	72.2% (57/79)	78.4% (80/102)	80.0% (32/40)	76.2% (80/105)
	*p*-value	0.329		0.625	
24 h sICH	Yes	12.7% (10/79)	0% (0/102)	27.5% (11/40)	7.6% (8/105)
	No	87.3% (69/79)	100% (102/102)	72.5% (29/40)	92.4% (97/105)
	*p*-value	<0.001		0.002	
**Outcomes**					
90D mortality	Yes	46.4% (39/84)	0% (0/102)	42.5% (17/40)	0% (0/105)
	No	53.6% (45/84)	100% (102/102)	57.5% (23/40)	100% (105/105)
	*p*-value	<0.001		<0.001	

mRS indicates modified Rankin Scale; NIHSS indicates National Institute of Health Stroke Scale; ASPECT indicates Alberta Stroke Program Early CT Score; TOAST indicates Trial of ORG 10172 in Acute Stroke Treatment; OTA indicates onset time to admission; END indicates early neurological deterioration; ICH indicates intracranial hemorrhage; sICH indicates symptomatic intracranial hemorrhage; OTA indicates time from onset to admission; OTP indicates time from onset to puncture; OTR indicates time from onset to reperfusion.

**Table 2:** Multivariable analysis of risk factors associated with 90-day poor outcomes (mRS scores of 4 to 6) following mechanical thrombectomy in patients with acute anterior circulation large-vessel occlusion.

Variables	Training set (90D mRS ≥ 4, *n* = 84)		Validation set (90D mRS ≥ 4, *n* = 40)	
	OR (95%CI)	*p*-value	OR (95%CI)	*p-*value
Age	1.049 (1.016–1.083)	0.003**	1.051 (1.008–1.095)	0.020*
Glucose	1.163 (1.038–1.303)	0.009**	1.062 (0.945–1.1923)	0.311
NIHSS	1.066 (0.995–1.142)	0.069	1.187 (1.055–1.335)	0.004**
Unsuccessful recanalization	3.730 (1.688–8.245)	0.001**	2.537 (0.956–6.731)	0.062
END	3.383 (1.411–8.106)	0.006**	3.548 (1.068–11.787)	0.039*

NIHSS indicates National Institute of Health Stroke Scale; END indicates early neurological deterioration.*p-value <0.05, **p-value <0.01.

### Construction of the nomogram for the prediction of 90-day poor outcomes in patients undergoing MT and its performance validation

**Figure 3:**
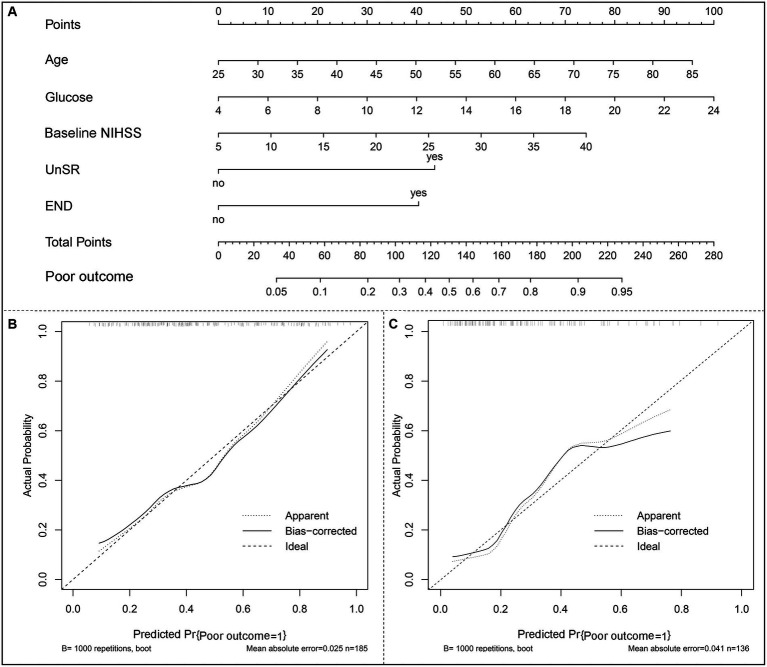
The nomogram model of a 90-day poor outcome (mRS scores of 4–6) in LVO patients after mechanical thrombectomy. **(A)** A nomogram for predicting the probability of a 90-day poor outcome. **(B)** A calibration curve of the training set. **(C)** A calibration curve of the validation set. The calibration plot with bootstraps of 1,000 repetitions represents the degree of fit between actual and nomogram-predicted poor outcomes. The x-axis represents the predicted probability of a 90-day poor outcome. The y-axis indicates the actual rate of 90-day poor outcomes. The dashed line represents the reference line, where an ideal nomogram would be. The dotted line represents the performance of the nomogram, while the solid line is corrected for any bias in the nomogram.

**Figure 4:**
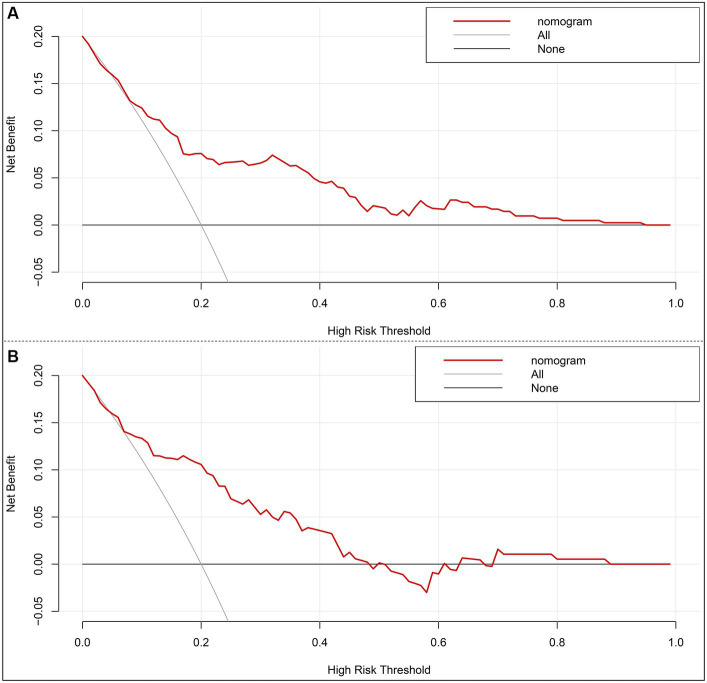
Decision curve analysis of the nomogram in the training set **(A)** and the validation set **(B)**. *x*-axis, the threshold probability; *y*-axis, the net benefit. The gray line indicates that all acute ischemic stroke patients undergoing mechanical thrombectomy will develop poor functional outcomes at 3 months. The black line indicates that no acute ischemic stroke patients undergoing mechanical thrombectomy will develop poor functional outcomes at 3 months. The red line is the nomogram to predict 3-month poor functional outcomes in AIS patients undergoing mechanical thrombectomy.

A nomogram model based on the results of multivariable regression analysis was developed to predict the 90-day poor outcome by assigning each independent predictor a score ranging from 0 to 100. The cumulative sum of the contribution scores for each factor in the nomogram represents the probability of a 90-day poor outcome (range, 0–280 points; Figure 3A). The performance of the nomogram was validated by calculating the C-index, which was 0.763 (95% CI: 0.693–0.832) and 0.804 (95% CI: 0.719–0.889) in the training and the validation sets, respectively. A calibration plot with bootstraps of 1,000 repetitions was employed to compare the agreement of the 90-day poor outcome between nomogram predictions and actual observations, which revealed that the nomogram had good predictive accuracy in both the training and validation sets (Figures 3B,C). A decision curve analysis was performed to evaluate the net benefit of the nomogram model in predicting 90-day poor outcomes after MT in LVO patients. Figure 4 shows that, when the risk threshold ranged from 14 to 98% and from 7 to 92% in the training and in the validation sets, respectively, using the nomogram model in the current study to predict 90-day poor outcomes in stroke patients after MT resulted in a greater net benefit than either all AIS patients undergoing MT or no patients undergoing MT. For example, if the personal threshold probability of a patient was 50%, then the net benefit in this nomogram model was 14.6% (95% CI: 8.6–25.4%) and 5.9% (95% CI: 0–15.4%) in the training and the validation sets, respectively.

### Translation of the nomogram model into practice

**Figure 5:**
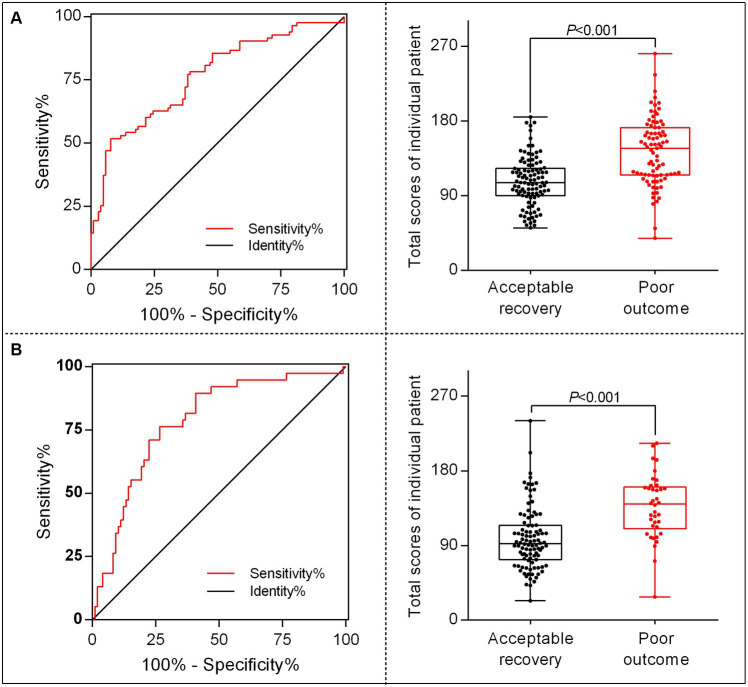
The receiver operating characteristic (ROC) curve and scatter plot of total scores in individual patients translated by the nomogram calculator. The ROC for identifying the optimal threshold (left) and the scatter plot of the total scores (right) in AC-LVO patients undergoing MT in the training set **(A)** and in the internal validation set **(B)**. AC-LVO, anterior circulation large-vessel occlusion; MT, mechanical thrombectomy.

To better translate the nomogram into clinical application, we developed a calculator for calculating each patient’s total score based on the contributions of each indicator in the model; thus, 96 points were assigned for patient ages ranging from 25 to 85 years, 100 points for glucose levels ranging from 4.0 to 24 mmol/L, 74 points for baseline NIHSS scores ranging from 5 to 40 score, 44 points for unsuccessful recanalization, and 40 points for the occurrence of END. The total score for individual patients was then calculated as follows: total score = (age–25) × [96/(85–25)] + (glucose level–4) × [100/ (24–4)] + (baseline NIHSS score–5) × [74/ (40–5)] + (0 or 1 of unsuccessful recanalization) × 44 + (0 or 1 of END) × 40. Then, we identified the optimal cutoff value for differing 90-day poor outcomes after MT in patients with AC-LVO, which was 144.65 in the training set and 110.72 in the validation set, showing a sensitivity of 51.8% (training set) and 76.3% (validation set) and a specificity of 92.2% (training set) and 73.5% (validation set). Furthermore, the chances of developing poor outcomes were approximately 60% in the training set and 35% in the validation set. The receiver operating characteristic curves and scatter plots of the total scores of the patients in the training and validation sets are shown in Figure 5.

## Discussion

The present study developed a nomogram model composed of age, glucose level, baseline NIHSS score, unsuccessful recanalization, and END to predict the probability of a poor outcome at 90 days after MT in patients with AC-LVO. This prognostic nomogram for the prediction of 90-day poor outcomes was derived from a prospective, multicenter, randomized clinical trial of MT with two flow reconstruction devices within 8 h of symptom onset in AIS. Additionally, it was externally validated in a retrospective cohort study from another single center. The nomogram showed good predictive power with C-index values of 0.763 (0.693–0.832) in the training set and 0.804 (0.719–0.889) in the validation set.

In the present study, 45.2% (84/186) of the patients developed poor outcomes (mRS scores of 4–6) at 90 days after MT, which was comparable to the corresponding values (37.1%) in the highly effective reperfusion evaluated in the multiple endovascular stroke (HERMES) clinical trial (9) and the individual patient data meta-analysis on the RCTs of MT across a 6–24 h time window (AURORA trial; 40%) (17). The incidence of poor outcomes (mRS scores of 4–6) at 90 days after MT in AC-LVO patients documented by the previous RCTs varied substantially (6–8), ranging from 12 to 48.79%, which was expected to be further reduced by actively screening the relevant risk factors of prognosis. In addition, we achieved a successful recanalization rate of 76.3%, consistent with the 71% rate in the HERMES trial (9) and 81% in the AURORA clinical trial (17). With the sICH rate of 5.4% and a mortality rate of 20.9% being comparable to those in the HERMES (4.4 and 15.3%, respectively) (9) and AURORA (5.3 and 16.5%, respectively) trials (17), our study confirmed that MT was safe for treating AIS patients with AC-LVO within 8 h of symptom onset.

At present, scoring systems (13, 14, 18–24) and nomograms (16, 25, 26) are the two main types of prognostic prediction tools for accurately and conveniently predicting clinical outcomes after MT. Although the existing scoring systems, including the THRIVE score (20), PRE score (14), and MR PREDICT score (27), have been externally validated, most of them focused on the 90-day independent functional outcomes (mRS scores of 0–2) or unfavorable outcomes (mRS ≥ 3), with less focus on the 90-day poor outcomes (mRS ≥ 4) (Supplementary Table S2) (12, 18, 19, 21, 22, 28). Among these, HAIT (18) and HIAT2 (19) were designed for the prediction of poor outcomes (mRS scores of 4–6) at discharge, which may have undermined their efficiency in predicting longer prognoses in clinical practice. The subsequent SAD score (22) and mTHRIVE score (21) that incorporated radiology variables based on the previous PRE (14) and THRIVE scores (20) were effective when applied for the prediction of 90-day poor outcomes; nevertheless, population-based cohort trials in large sample sizes were required to validate the utility of these scoring tools in clinical practice. Most of the recently developed nomogram models for predicting the 90-day outcomes after MT in acute AC-LVO patients were focused on either a good outcome (mRS scores of 0–2) or an unfavorable outcome (mRS scores ≥3). To our knowledge, the nomogram model constructed in this study is the first of its kind for the prediction of 90-day poor outcomes (mRS scores of 4 to 6) after MT in patients with AC-LVO, which provided a more accurate individualized estimation of the risk probability of poor outcomes (ranging from 0.2 to 0.95) by translating the nomogram into a formula instead of tracing lines along the scheme.

Prognostic prediction models have been classified into pre-procedure and post-procedure tools (11), which would be helpful for the selection and management of thrombectomy patients in decision-making. However, the variables used by scoring systems and nomogram models for predicting the risk of unfavorable outcomes vary considerably (12), posing a dilemma for their clinical application. In our study, three pre-treatment parameters (age, baseline NIHSS score, and glucose level on admission) were incorporated into the nomogram model. Previous studies have reported that advanced age is strongly associated with poor clinical outcomes in patients receiving MT (29–31). In the PRE scoring system, it was also reported that, when applied to assess a good prognosis for octogenarian patients who have successfully achieved recanalization, the requirement was for their PRE scores to be within a specific range (14). Unlike previous studies that converted ages into a categorical variable by dichotomizing them on the basis of the median value, our study included age as a continuous variable in the nomogram and reported a contribution of 95 points to the total score to ensure complete utilization of within-category information. The baseline NIHSS score is a well-recognized variable that has been shown to be associated with 90-day functional independence following MT in AC-LVO (14, 19, 23). In our nomogram model, the baseline NIHSS score contributed 74 points to the total score for patients predicting the 90-day poor outcomes after MT in acute AC-LVO patients, although it was not an independent predictor of poor outcomes in the final nomogram model. The mechanisms by which hyperglycemia damages the blood vessels, causes the accumulation of extracellular glutamate, disrupts the blood–brain barrier, and eventually aggravates brain injury have been well established (32–34). Therefore, higher levels of glucose would exacerbate the clinical outcomes in patients with LVO. Kim et al. (35) and Goyal et al. (36) demonstrated that hyperglycemia increased the risk of poor outcomes of incomplete reperfusion after MT, supporting the synergistic effect of ischemia–glucose concentration. Similarly, our study found that the glucose level on admission significantly contributed to a higher risk of poor outcomes in LVO patients after MT in the prognostic nomogram model, with 100 points contributing to the total score of the nomogram model. Consistently, the meta-analysis conducted by Calos et al. (37) also demonstrated that hyperglycemia could increase the risk of unfavorable clinical outcomes in LVO patients undergoing MT.

Unlike the previous scoring systems and the nomogram models based on pre-treatment variables for prognostic prediction, we developed a nomogram that also incorporated two post-treatment variables for predicting the probability of 90-day poor outcomes in AIS patients eligible for MT. Although the reasons why 19–43% of AIS patients with futile recanalization exhibited unfavorable outcomes (23) and 34% of AIS patients with failed recanalization obtained favorable outcomes (38) have not been fully elucidated, the beneficial effect of MT in AIS patients remains undeniable. Nevertheless, an unsuccessful recanalization rate of 12–41.3% (8) has been associated with the unfavorable outcome of AIS patients after MT, weakening the clinical efficacy of MT to a certain extent. In the IER-START nomogram (16), the reperfusion grade was demonstrated as a strong post-treatment radiological predictor of unfavorable outcomes 3 months after thrombectomy. The most recently published study of a clinical nomogram also reinforced the increased risk of 3-month unfavorable outcomes after unsuccessful recanalization (25). Consistently, we found that unsuccessful recanalization was a strong predictor of poor clinical outcomes, with a contribution of 44 to the total score, in patients with AIS following MT and can be effectively used to estimate the probability of poor outcomes after MT. The incidence of END in LVOS patients after MT is 35–42% (39–41), and it has been identified as a definite risk factor for 90-day poor clinical outcomes in AIS patients treated with MT (40, 42–44). Unfortunately, no previous study has reported its role in predicting the probability of unfavorable outcomes in AIS patients treated with MT. We employed END as a risk factor that exhibited strong power in predicting the probability of a 90-day poor outcome, with a contribution of 40 points to the total score.

### Limitations

This study had some limitations. First, the sample sizes of both the training and validation sets for model construction and validation were relatively small. Therefore, studies with larger sample sizes are required for future validation. Second, the data used for our nomogram model were derived from randomized controlled clinical trials, which may not be the optimal design for prognostic studies. Unlike those in real-world studies, RCTs enroll patients who strictly meet the inclusion criteria and provide informed consent; thus, the study population in an RCT is not fully representative of the general population. The generalizability of the model may have been weakened by this factor.

## Conclusion

Our post-treatment nomogram model consisting of five variables (age, baseline NIHSS score, glucose level on admission, unsuccessful recanalization, and END) exhibited very good efficacy in external validation and can be used to accurately estimate the probability of a poor outcome at 90 days for LVOS patients who underwent MT within 8 h after the stroke onset. This post-MT nomogram model can be easily translated into practice and can complement existing models.

## Data availability statement

The raw data supporting the conclusions of this article will be made available by the authors, without undue reservation.

## Ethics statement

The studies involving humans were approved by the ethics committee of PLA Rocket Force Characteristic Medical Center (X2017008). The studies were conducted in accordance with the local legislation and institutional requirements. The participants provided their written informed consent to participate in this study.

## Author contributions

XL: Writing – original draft, Data curation, Investigation. CL: Writing – original draft, Writing – review & editing, Investigation, Methodology, Project administration, Resources. A-fL: Writing – original draft, Writing – review & editing, Investigation, Methodology. C-cJ: Investigation, Writing – review & editing, Methodology, Project administration. Y-qZ: Investigation, Methodology, Writing – review & editing. YL: Investigation, Methodology, Writing – review & editing. Y-yZ: Investigation, Writing – review & editing, Formal analysis. H-yL: Formal analysis, Writing – review & editing, Software. W-jJ: Writing – review & editing, Conceptualization, Funding acquisition, Methodology, Resources, Supervision. JL: Conceptualization, Supervision, Writing – review & editing, Formal analysis, Writing – original draft.

## Glossary

AIS: Acute ischemic stroke
AC: Anterior circulation
LVO: Large-vessel occlusion
MT: Mechanical thrombectomy
RCTs: Randomized clinical trials
PRE: Pittsburgh response to endovascular therapy
THRIVE: Totaled health risk in vascular events
HIAT: Houston intra-arterial therapy
IER-START: Italian Registry of Endovascular Stroke Treatment in Acute Stroke
TVSS: Tor Vergata Stroke Score
HERMES: Highly effective reperfusion using multiple endovascular devices
IV rtPA: Intravenous recombinant tissue plasminogen activator
mRS: Modified Rankin Scale
NIHSS: National Institute of Health Stroke Scale
DSA: Digital subtraction angiography
ASPECT: Alberta Stroke Program Early Computer Tomography
mTICI: modified thrombolysis in cerebral infarction
HBC: Heidelberg Bleeding Classification
PH: Parenchymal hematoma
ICH: Intracranial hemorrhage
sICH: Symptomatic intracranial hemorrhage
ROC: Receiver operating characteristic
IC-ICA: intracranial segment of internal carotid artery
END: early neurological deterioration
