# Supplementary material for: Application of a nomogram model for the prediction of 90-day poor outcomes following mechanical thrombectomy in patients with acute anterior circulation large-vessel occlusion
Source: Front Neurol. 2024 Jan 19;15:1259973. doi: 10.3389/fneur.2024.1259973 (PMC10836145; doi:10.3389/fneur.2024.1259973)
Supplement: Supplementary file 1 [file Data_Sheet_1.docx]

Supplementary Figure 1





Figure legend

Supplementary Figure 1. Kaplan-Merier survival curve of patients with (A) successful recanalization v.s. unsuccessful recanalization, (B) END v.s. no-END, (C) sICH v.s. no-sICH, (D) 90-day severe disability (mRS scores of 4 to 6) v.s. acceptable functional recovery (mRS socres of 0 to 3). SR, successful recanalization; UnSR, unsuccessful recanalization; END, early neurological deterioration; sICH, symptomatic intracerebral hemorrhage.

Supplementary Table 1 Baseline characteristics of patients undergoing mechanical thrombectomy

| Variables | | % (n); IQR (N=187) |
| --- | --- | --- |
| Demographics | |  |
| Age (years) (median, IQR) | | 66 IQR (58～74) |
| ≤66 | | 52.4% (98/187) |
| >66 | | 47.6% (89/187) |
| Sex | |  |
| male | | 56.7% (106/187) |
| female | | 43.3% (81/187) |
| Medical history | |  |
| Smoke habits | | 27.3% (51/187) |
| Hypertension | | 59.9% (112 /187) |
| Diabetes mellitus | | 21.9% (41 /187) |
| Coronary heart disease | | 16.6% (31/187) |
| Atrial fibrillation | | 48.1% (90 /187) |
|  | | 27.3% (51/187) |
| Previous Stroke | | 11.8% (22 /187) |
| Baseline data | |  |
| Pre mRS Score | |  |
| =0 | | 94.1% (176 /187) |
| ≠0 | | 5.9% (11/187) |
| Glucose(mmol/L) (median, IQR) | | 6.91 IQR (5.91～8.9) |
| ≤6.91 | | 49.7% (93/187) |
| >6.91 | | 50.3% (94/187) |
| Systolic Blood Pressure(mmHg) | | 140 IQR (124～159) |
| ≤140mmHg | | 54% (101/187) |
| >140mmHg | | 46% (86/187) |
| Diastolic Blood Pressure(mmHg) | | 82 IQR (75～90) |
| ≤82mmHg | | 53.5% (100/187) |
| >82mmHg | | 46.5% (87/187) |
| Glucose(mmol/L) | | 6.91 IQR (5.91～8.9) |
| ≤6.91 | | 49.7% (93/187) |
| >6.91 | | 50.3% (94/187) |
| Baseline NIHSS (median, IQR) | | 14.0 IQR (11～18) |
| <14 | | 44.4% (83/187) |
| ≥14 | | 55.6% (104/187) |
| ASPECT (median, IQR) | | 9 IQR (8～10) |
| >9 | | 34.2% (64/187) |
| ≤9 | | 65.8% (123/187) |
| TOAST | Atheroma | 21.9% (41/187) |
| Cardioembolic | | 78.1% (146/187) |
| Intravenous thrombolysis | | 32.6% (61/187) |
| General anesthesia | | 47.6% (89/187) |
| Occlusion location | |  |
| M1 | | 71.7% (134 /187) |
| ICA | | 28.3% (53/187) |
| Unsuccessful recanalization | | 24.1% (45/187) |
| Residual severe stenosis | | 17.6% (33 /187) |
| Remote embolization | | 23% (43/187) |
| Rescue therapy | | 24.1% (45/187) |
| Anticoagulant therapy | | 56.7% (106/187) |
| OTA (median, IQR) | | 186 IQR (117～270) |
| ≤180 | | 48.7% (91 /187) |
| >180 | | 49.7% (93 /187) |
| OTP (median, IQR) | | 304 IQR (227～380) |
| ≤304 | | 50.3% (94/187) |
| >304 | | 49.7% (93/187) |
| OTR (median, IQR) | | 413 IQR (324～495) |
| ≤413 | | 50.3% (94/187) |
| >413 | | 49.7% (93/187) |
| Complications | |  |
| END | | 19.3% (36/187) |
| 24h ICH | | 31% (58/187) |
| 24h sICH | | 5.3% (10/187) |
| Outcomes | |  |
| 90 Days mRS | |  |
| mRS ≤ 3 | | 54.8% (102 /186) |
| mRS ≥ 4 | | 45.2% (84/186) |
| Censored | | 0.5% (1/187) |
| 90D mortality | | 21.0% (39/186) |

MT, mechanical thrombectomy; NIHSS, National Institute of Health stroke scale; ASPECT, Alberta Stroke Program Early CT Score; M1, Middle cerebral artery 1; mRS, modified Rankin Scale; ICH, intracranial hemorrhage; sICH, symptomatic intracranial hemorrhage; END, early neurological deterioration; OTA, time from onset to admission; OTP, time from onset to puncture; OTR, time from onset to reperfusion; NA, not applicable.

Supplementary Table 2 Overview of scoring system and nomogram for the prediction of 90-day poor outcome (mRS of 4-6) in patients with AC-LVO underwent mechanical thrombectomy.

|  | Sources  Author/year | Study type and period | Intervention | Time window | Sample size | Endpoint Incidence | AUC | Points of the score | Predictors for 90-day poor outcome (mRS of 4-6) |
| --- | --- | --- | --- | --- | --- | --- | --- | --- | --- |
| 1 | Hallevi et al. 2009[18]. HIAT score | Retrospective study  1998-2007 | IAT | ≤6 | N=190 | On discharge:  66.3% (126/190) | 0.730 | Age: >75=1;  NIHSS: >18=1;  Glucose: >150mg/dL=1; | Age: OR 1.028 [0.877-0.959], *P*=0.049;  Baseline NIHSS: OR 1.084 [1.453-14.477], *P*=0.013;  Glucose: OR 1.011[1.107-4.058], *P*=0.013; |
| 2 | Sarraj et al. 2013 [19]. HIAT2 score | Retrospective cohort study. 2003.01-2011.05 | IAT | ≤8 | N=163 | On discharge:  44.2% (72/163) | 0.748 | Age (≤59=0, 60–79=2, ≥80 years=4);  Glucose (<150=0, ≥150=1);  NIHSS (≤10=0, 11–20=1, ≥21=2);  ASPECT (8–10=0, ≤7=3); | HIAT2 score ≥5: OR 5.88[1.96-17.64], *P*=0.02; |
| 3 | Liggins et al. 2015[22]. SAD score | Retrospective analysis on DEFUSE 2  2008.07-2011.09 | EVT | ≤12 | N=110 | 90 D:  40.9% (45/110) | 0.820 | Pre-EVT(SADscore）  DWI volume (≤15cc=0, ＞15cc=1);  Age(≤55=0,56-69=1,70-79=2, ≥80=3); | Age: P < 0·001; β = 0·087;  DWI volume: P = 0·023; β =0·025; |
| 4 | Ryu et al. 2019 [21]. mTHRIVE score | Retrospective mmutlicenter registry  2010.09-2015.12 | EVT | ≤10 | N=482 | 3-month:  38% (183/482) | 0.771 | z=x+a×y  x: is the given predictive score (HAIT2, THRIVE, or PRE score);  y: is the collateral grade;  a: is a constant integer, to decide the most predictable equation. | (HIAT2, THRIVE, PRE), collateral grades |
| 5 | Karamchandani, et al. 2022 [28]. CLEOs | Retrospective study  2016.11-2020.07 | EVT | ≤24 | N=453 | 90-day:  D: 39.3% (178/453)  V: 42.5% (82/193) | 0.75 | CLEOS=(5×Age)+(10×NIHSS)+Glucose-(150×Cerebral Blood Volume Index) | CLEOS (per 1-point increase): OR 1.0088 [1.006-1.010], *P* <0.001;  Age: OR 1.039 [1.023-1.056], *P* <0.001;  NIHSS: OR 1.078 [1.041-1.117], *P* <0.001;  Glucose: OR 1.008 [1.003-1.012], *P* =0.0003;  CBV Index: OR 0.316 [0.082-1.224], *P* =0.0954; |

IAT, intra-arterial recanalization therapy; EVT, endovascular therapy; HIAT, Huston IAT; DEFUSE 2, diffusion and perfusion imaging evaluation for understanding stroke evolution 2; SAD, the Stanford Age and DWI; THRIVE, totaled health risks in vascular events; PRE, Pittsburgh response to endovascular therapy; CLEOS, Charlotte Large occlusion Endovascular therapy Outcome Score; D, derivation; V, validation; NIHSS, National Institutes of Health Stroke Scale; ASPECT, Alberta Stroke Program Early CT score; DWI, diffusion-weighted imaging;
